# Role of the Atypical MAPK ERK3 in Cancer Growth and Progression

**DOI:** 10.3390/cancers16071381

**Published:** 2024-03-31

**Authors:** Lobna Elkhadragy, Amanda Myers, Weiwen Long

**Affiliations:** 1 Department of Biochemistry and Molecular Biology, Boonshoft School of Medicine, Wright State University, Dayton, OH 45435, USA; lobna@uic.edu (L.E.); myers.299@wright.edu (A.M.); 2 Department of Radiology, University of Illinois at Chicago, Chicago, IL 60612, USA

**Keywords:** MAPK, ERK3, kinase signaling, post-translational modifications (PTMs), cancer, cell growth, cell migration and invasion, tumor development and progression

## Abstract

**Simple Summary:**

Extracellular signal-regulated kinase 3 (ERK3) is a member of the atypical mitogen-activated protein kinase (MAPK) subfamily. While it remains largely unknown regarding the upstream stimuli and regulators for its kinase activation, ERK3 has been shown to play important roles in physiological processes and cancers. This review discusses the growing body of work that unveils the roles for ERK3 in regulating cell growth, migration and invasion, as well as cancer growth and progression. In addition, it provides important insights on the molecular regulation of ERK3 expression and activity and its implications in different signaling pathways in cancers.

**Abstract:**

Extracellular signal-regulated kinase 3 (ERK3) is an atypical mitogen-activated protein kinase (MAPK) whose structural and regulatory features are distinct from those of conventional MAPKs, such as ERK1/2. Since its identification in 1991, the regulation, substrates and functions of ERK3 have remained largely unknown. However, recent years have witnessed a wealth of new findings about ERK3 signaling. Several important biological functions for ERK3 have been revealed, including its role in neuronal morphogenesis, inflammation, metabolism, endothelial cell tube formation and epithelial architecture. In addition, ERK3 has been recently shown to play important roles in cancer cell proliferation, migration, invasion and chemoresistance in multiple types of cancers. Furthermore, accumulating studies have uncovered various molecular mechanisms by which the expression level, protein stability and activity of ERK3 are regulated. In particular, several post-translational modifications (PTMs), including ubiquitination, hydroxylation and phosphorylation, have been shown to regulate the stability and activity of ERK3 protein. In this review, we discuss recent findings regarding biochemical and cellular functions of ERK3, with a main focus on its roles in cancers, as well as the molecular mechanisms of regulating its expression and activity.

## 1. Introduction

Mitogen-activated protein kinases (MAPKs) are a family of Ser/Thr kinases that play essential roles in the transduction of extracellular signals into a variety of intracellular responses. Based on their structural and regulatory features, MAPKs are classified as conventional and atypical MAPKs [1,2]. The conventional MAPKs include extracellular signal-regulated kinases 1/2 (ERK1/2), p38 isoforms (α, β, γ and δ), c-Jun amino-terminal kinases 1/2/3 (JNK1/2/3) and ERK5. These protein kinases harbor a conserved TXY motif in their activation loops and their activation pathways comprise a cascade of three phosphorylation events exerted sequentially by a MAPK kinase kinase (MAPKKK), a MAPK kinase (MAPKK) and the effector MAPK itself. The atypical MAPKs include ERK3/4/7/8 and Nemo-like kinase. With the exception of ERK7/8, atypical MAPKs have a single phosphorylation site in their activation motifs instead of a TXY motif [3]. In addition, the activation pathways of atypical MAPKs are not organized into three-tiered kinase cascades as identified in the conventional MAPKs.

**Figure 1:**
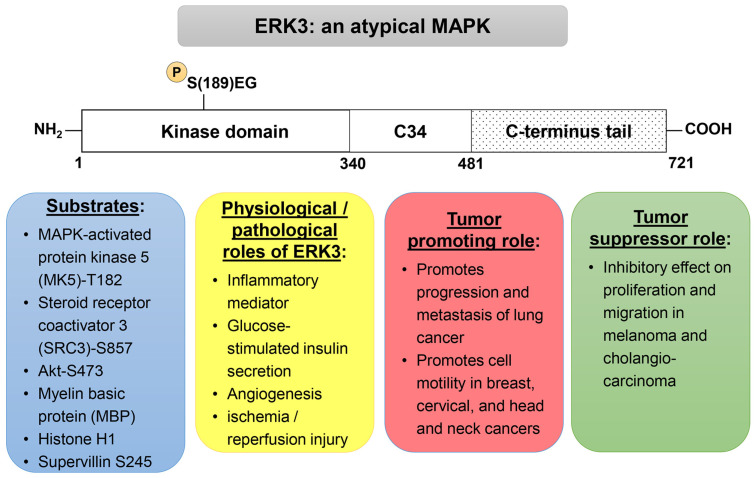
Overview of ERK3 protein structure and functions. SEG is the activation motif in which serine 189 (S189) is the phosphorylation site. C34 stands for the conserved domain in ERK3 and ERK4.

ERK3 (official gene name: *MAPK6*) was identified in the early 1990s through homology screening of a rat brain cDNA library using an ERK1-derived probe [4]. Soon after that, the human ERK3 gene was cloned and characterized [5,6]. The human ERK3 protein comprises 721 amino acids that form three major domains/regions. The kinase domain of ERK3 lies in the amino terminus and is nearly 45% identical to that of ERK1. The kinase domain is followed by a region that is highly conserved between ERK3 and ERK4 and hence called C34. Beyond the C34 region, there is an unusually long carboxyl-terminus tail (Figure 1) [3,5,6,7]. ERK3 has a single phospho-acceptor site in its SEG motif within the activation loop that is not phosphorylated by conventional MAPKKs (Figure 1) [3]. While most MAPKs are expressed in all eukaryotes, ERK3 and ERK4 are expressed only in vertebrates. ERK3 mRNA is ubiquitously expressed in adult mouse, rat and human tissues, although its relative expression level is variable among tissues [4,5,6,7]. In adult humans, the highest levels of ERK3 mRNA are found in the skeletal muscles and the brain.

In contrast to conventional MAPKs, for which a large number of substrates have been identified, ERK3 does not phosphorylate many generic MAPK substrates in vitro, such as c-Jun, Tal1, MyoD, c-Elk or papilloma virus protein E7, and the number of currently known bona fide substrates is small (Figure 1) [3,8]. The kinase domain of ERK3 expressed in bacteria exhibits very weak kinase activity towards substrates, although it autophosphorylates, mainly on the S189 residue of the activation motif [8]. Greater activity is observed for ERK3 expressed in Sf9 insect cells or in cultured mammalian cells, possibly due to post-translational modifications, and these purified ERK3 proteins efficiently phosphorylate the MAPK substrates myelin basic protein (MBP) and histone H1 in vitro [5,9].

The downstream targets of ERK3 remained mysterious for a long time following its identification, until MAPK-activated protein kinase 5 (MK5), a member of the MAPK-activated protein kinase (MAPKAPK) family, was revealed as a downstream target of ERK3 simultaneously by two groups of researchers [10,11]. In light of this and the spatiotemporal coexpression of MK5 and ERK3 during development and in adult tissues [10,11], it is very likely that the ERK3/MK5 signaling module has important physiological roles. Both the groups demonstrated that ERK3 and MK5 interact with each other and co-translocate from the nucleus to the cytoplasm, which leads to the stabilization of ERK3 protein. While both groups agreed that ERK3 stimulates the phosphorylation and activity of MK5, they proposed different mechanisms for this process. The work of Seternes and colleagues showed that ERK3 activates MK5 by phosphorylating its activation loop at Thr182 [10]. On the other hand, Schumacher and colleagues demonstrated that through physical interaction, ERK3 promotes the autophosphorylation of MK5 at the same residue (Thr182), leading to its activation [11]. The interaction of ERK3 with MK5 is mediated by both S189 within the activation motif and by the FRIEDE motif in the C-terminal lobe of the kinase domain of ERK3. A single point mutation of S189 in the SEG activation motif or I334 in the FRIEDE motif abrogates the interaction of ERK3 with MK5 [12,13].

## 2. Physiological Functions of ERK3

Recent work has provided advancements in our understanding of the physiological roles ERK3 may play at the organismal and cellular level. ERK3 plays a role in multiple processes, including pathways of inflammation, metabolism and obesity, angiogenesis and dendritic spine formation (Figure 1).

While initial work suggested knockout of ERK3 in C57BL/6 mice resulted in neonatal death due to pulmonary functional defects caused by impaired lung maturation, the group later determined this was likely due to off-target effects caused by changes to the transcription of neighboring genes [14,15,16]. Similarly, although initial studies suggested a role for ERK3 in T-cell and thymocyte activation and maturation [17,18,19], newer mouse models do not support this. Interestingly, however, overall post-natal growth in mice was significantly decreased when ERK3 was knocked out or a kinase-dead ERK3 was knocked in [20]. This suggests ERK3 does have observable physiological roles that require its kinase activity.

Recent studies suggest ERK3 plays a role in inflammatory signaling pathways. Work published in 2014 demonstrated treatment with TNFα and IL-1β doubled ERK3 expression level at 6 h in human umbilical vein endothelial (HUVEC) cells [21]. Later work by another group confirmed ERK3 level was increased by IL-1β in human choroid plexus endothelial cells [22]. ERK3 knockdown experiments found this protein is important for the expression of CXCL8 (IL-8), CXCL2, CXCL10 and CXCL6 in human colonic primary epithelial cells treated with lipopolysaccharides. This effect was shown to occur at the transcript level with a decrease in IL-8 promotor activity with knockdown of ERK3. Further, a study published in 2023 noted ERK3 level was significantly increased with other inflammatory-related proteins when the membrane palmitoylated protein 2 (MPP2) was mutated to MPP2 K315N [23]. This process appears to require annexin A2 (ANXA2), a protein with roles in membrane trafficking and cell metastasis [24], as silencing ANXA2 reduced ERK3 protein level and alleviated MPP2-N315-induced experimental autoimmune uveoretinitis (EAU) inflammation.

In addition, ERK3 has been shown to be implicated in inflammation and ischemic-reperfusion (I/R) injury. Myocardial ischemia/reperfusion injury modeled in H9C2 cells (a rat embryonic heart cell line) resulted in increased ERK3 protein level [25]. This study also found the microRNA miR-374a-5p directly targets the 3′ untranslated region of ERK3 mRNA and imparts a protective effect on cells, which could be significantly diminished by overexpression of ERK3. Another study similarly found a long non-coding RNA, NEAT1, contributed to I/R injury by binding to miR-459-3p and preventing its targeting of ERK3 [26]. This effect was also seen in a rat model of hepatic I/R injury and hepatic cell lines subjected to hypoxia and re-oxygenation [27]. While propofol, a drug which may have a protective effect in some I/R injuries, reduced both apoptosis and the expression of ERK3, this rescue effect was negated after overexpression of ERK3. In an induced cerebral I/R injury cell model using the neuroblastoma cell line SH-SY5Y, expression of ERK3 was initially reduced but at 24 h had significantly increased compared with the normal control group [28]. Taken together, these studies suggest that ERK3 expression level and/or activity are upregulated upon the induction of I/R injury and contribute to I/R injury, possibly by promoting cell inflammation, oxidative stress and death.

Moreover, research is uncovering a metabolic role for ERK3. The knockdown of ERK3 in rat pancreatic islets abolished glucose-stimulated insulin secretion, suggesting ERK3 is important in the process of insulin secretion [29]. In differentiated 3T3L1 cells, an adipocyte-like mouse fibroblast cell line, β-adrenergic receptor stimulation led to ERK3 stabilization in a protein kinase A (PKA)-dependent manner and ERK3/MK5 activation of forkhead box O1 (FOXO1), leading to nuclear translocation [30]. Knockdown of ERK3 results in decreased transcription of certain genes, including *Atgl*, and the inhibition of lipolysis in mouse adipose tissue coupled with reduced weight gain, reduced glucose levels and increased energy expenditure. Continued work showed ERK3 and MK5 levels are increased in subcutaneous adipose tissues of obese humans compared with lean counterparts and deleting ERK3 expression using a tamoxifen-inducible deletion mouse model resulted in less weight gain compared with control animals when fed a high fat diet [31].

The ERK3/MK5 complex also regulates neuronal cytoskeleton complexes and increases neuronal dendritic spine formation in primary hippocampal neurons [9]. These functions are dependent on Septin7, a GTP-binding protein that is involved in various structural re-modelling processes of cells [32]. ERK3, through its C-terminus tail, interacts with Septin7, resulting in the formation of an ERK3/MK5/Septin7 ternary complex. Both ERK3 and MK5 possibly regulate the activity of Septin7 through phosphorylation of Binder of Rho GTPases (Borg) proteins, which are known to interact with septins and regulate their activity [9].

In endothelial cells, ERK3 promotes migration, proliferation and angiogenesis through upregulating vascular endothelial growth factor receptor 2 (VEGFR2), a primary VEGF receptor essential for endothelial cell function [21]. Similarly, human lymphatic endothelial cells over-expressing ERK3 had increased levels of VEGF-C, VEGFR-3 and TNF-α [33].

Besides the well-established effect on cell proliferation and migration, ERK3 also has been shown to play a role in cell differentiation. When nerve growth factor (NGF) is applied to PC-12 cells, (a rat pheochromocytoma cell line), ERK3 level is increased [34]. C2C12 cells (a mouse myoblast cell line) receiving differentiation media have increased ERK3 levels with a longer half-life. A later study confirmed USP20 de-ubiquitinates ERK3 to increase stability during C2C12 myoblast differentiation [35]. ERK3 kinase activity and phosphorylation of MK5 were then implicated in the modulation of mouse muscle growth and regeneration after injury. Mechanistically, ERK3 phosphorylates MK5, which phosphorylates FoxO3 to promote its degradation. This prevents high activation of MyoD and premature differentiation of C2C12 cells [14].

To date, the physiological functions identified for ERK3 are varied and many are poorly understood. Although some roles presently appear unrelated, future work to elucidate underlying mechanisms and pathways may lead to a more in-depth and unified understanding.

## 3. Roles of ERK3 in Cancer

The role of ERK3 in cancer progression has been a subject of significant interest. Studies have reported important and differential roles of ERK3 in different types of cancers.

### 3.1. Expression and Mutations of ERK3 in Cancers

An upregulation of ERK3 transcript and/or protein level was demonstrated in non-small cell lung cancers (NSCLCs) (in both lung adenocarcinomas and lung squamous cell carcinoma), chewing tobacco-induced oral squamous cell carcinoma, head and neck cancer and gastric cancer tissues as compared to normal tissues [36,37,38,39]. ERK3 protein also has heightened expression in actinic keratosis and cutaneous squamous cell carcinoma compared with normal skin tissue [40]. Additionally, in human lung adenocarcinoma, ERK3 mRNA expression is inversely correlated with PTEN protein expression [41].

Mutations in ERK3 have been detected in lung cancer, ovarian cancer and skin cancer tissues, albeit at very low frequency [42]. These mutations include R64C, L290V, L290P and G234C in the kinase domain, as well as K489T and E508K in the C34 domain of ERK3. Except for L290 mutations, shown to increase cytoplasmic localization and migration/invasion-promoting capability of ERK3 in cancer cells, little is known about the effects of other mutations of ERK3 on its functions in cancer cells.

### 3.2. Tumor-Promoting Roles of ERK3

#### 3.2.1. Cancer Cell Proliferation and Tumor Growth

**Figure 2:**
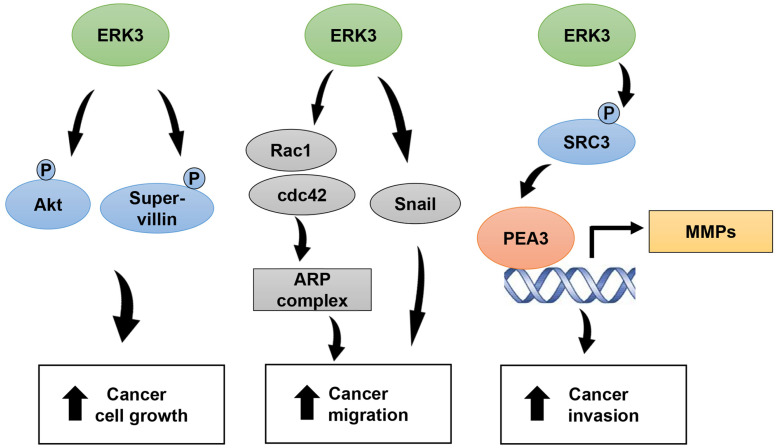
Molecular pathways underlying the role of ERK3 in promoting cancer cell growth and invasion. P in blue circle indicates phosphorylation.

Accumulating studies have suggested important roles for ERK3 in promoting cancer cell growth and tumor development in multiple types of cancers, including lung cancers, breast cancers and prostate cancers [43]. Of note, ERK3 plays varied roles in cancers, depending on cancer subtypes and genetic background. ERK3 silencing greatly reduced cell growth and/or anchorage-independent colony formation of KRAS^G12C^-positive H23 and H2122 NSCLC cell lines and xenograft tumor growth of the Calu-1 cell line also expressing KRAS^G12C^, whereas it had little effect on the growth of lung cancer cell lines H1299 and H1650 expressing wild-type KRAS [36,44]. KRAS activation (upon overexpression or G12 mutations) increased ERK3 phosphorylation at S189 within the activation motif [44]. Thus, it will be important to examine whether ERK3 S189 phosphorylation is increased in lung adenocarcinomas (LUADs) with KRAS G12 mutations versus LUADs expressing wild-type KRAS. Intriguingly, ERK3 expression level is upregulated in LUAD regardless of KRAS mutation status [44]. The idea that the role of ERK3 in cell growth is affected by other molecular alterations is supported by multiple lines of research, including a transgenic mouse study in which ERK3 was conditionally overexpressed in lungs [41]. ERK3 overexpression alone had little effect on lung epithelial cell growth (cell proliferation and apoptosis) and conditional deletion of *PTEN* tumor suppressor induced hyperplasia of lung epithelium. However, ERK3 overexpression, in combination with *PTEN* deletion, increased cell proliferation, decreased cell apoptosis and promoted lung tumor formation [41]. These findings suggest that ERK3 itself may not be able to transform normal epithelial cells but is capable of promoting tumor growth once cells are transformed and/or tumorigenic following the loss-of-function mutation of tumor suppressor genes (e.g., *PTEN*) or gain-of-function mutation(s) of oncogenes such as *KRAS*^G12^ mutations. In line with the cooperative role for ERK3 overexpression and *PTEN* deletion in promoting tumor growth in vivo [41], ERK3 was shown to activate AKT via phosphorylating S473, thereby promoting xenograft tumor growth of both lung cancer cells and breast cancer cells (Figure 2) [44]. The role of ERK3 in promoting tumor cell growth can be explained, at least partially, by its functions in regulating cell cycle progression. ERK3 is known to be involved in cell mitosis. ERK3 is hyper-phosphorylated in multiple sites of its C-terminus during mitosis, which leads to increased ERK3 protein stability [45]. Phosphorylated ERK3 was observed to colocalize with phospho-histone H3, a mitotic marker, in fetal lung and HeLa cervical cells [46]. An exact role for ERK3 in mitosis was revealed by a recent study: ERK3 interacts with and phosphorylates supervillin at S245 in mitosis and promotes myosin II activation and cytokinesis of breast cancer cells (Figure 2) [47]. In addition, ERK3 was shown to promote G1/S progression and proliferation of H1299 and A549 lung cancer cells [48]. Mechanistically, ERK3 knockdown led to a decrease in CDK2 and cyclin A levels and an increase in p21WAF1/Cip1 level. While the exact mechanism(s) remains to be explored, it might be related to the role of ERK3 in upregulating Akt signaling [43].

#### 3.2.2. Cancer Cell Migration, Invasion and Metastasis

Though ERK3 appears to have complex and subtle roles in cell proliferation and tumorigenesis, its role in tumor cell migration, invasion and metastasis is well established in many cancer types. Both knockdown and overexpression experiments have demonstrated that ERK3 promotes cancer cells’ migration and invasion. Transwell assays in multiple publications have solidified ERK3 as an enhancer of lung cancer cell motility [36,49,50,51]. Additional work has shown ERK3 promotes cancer cell mobility in breast, cervical and head and neck cancer cell lines [52,53,54,55]. Mechanistically, ERK3 promotes cancer cell migration and invasion through various mechanisms, including actin polymerization and cytoskeletal structure [55], Snail-mediated epithelial–mesenchymal transition (EMT) [56], IL-8-mediated chemotaxis [22] and matrix metalloprotein (MMP) gene expression (Figure 2) [36]. Interestingly, ERK3 achieves these by both kinase-dependent and -independent mechanisms [50].

First, ERK3 regulates cancer cell morphology and motility. In a recent study, ERK3 was associated with higher cell movement speed and Rac1/Cdc42 activity in human mammary epithelial cells (HMECs) (Figure 2) [57]. Depletion of ERK3 reduced Rac1 and Cdc42 interaction at the plasma membrane and reduced Rac1/Cdc42 interaction with the ARP-complex proteins ARP2, ARP3 and ARPC1A. Interestingly, the addition of ERK3 to a pyrene actin polymerization assay resulted in more efficient actin polymerization than ARP2/3 alone. In line with this, exogenous expression of ERK3 in breast cancer cells results in an increase in cell migration speed, decrease in cell spread area and rearrangements of the actin cytoskeleton [55]. These changes were proposed to be linked to the localization of ERK3 in the periphery of the cells. Interestingly, a catalytically inactive mutant of ERK3 decreased breast cancer cell spread area comparably to the effect of wild type ERK3, suggesting a kinase-independent function for ERK3 in modulating cellular morphology [55]. In agreement with this, ERK3 interacts with and stabilize SNAIL protein independent of its kinase activity, suggesting a new mechanism (Snail-mediated EMT) for ERK3′s role in modulating cell morphology and motility [56] (Figure 2).

In addition, ERK3 was shown to be a downstream target of Rab31, a Ras-associated GTP-binding protein that regulates protein transport between the Golgi/trans-Golgi network and plasma membrane/endosome [54]. Rab31 interacts with and stabilizes ERK3 and promotes migration and invasion through ERK3 in cervical cancer cell lines. Moreover, ERK3 upregulates c-Jun/AP1-mediated IL-8 gene transcription and promotes chemotaxis of tumor cells and leukocytes [22].

Besides its role in regulating cell morphology and motility, ERK3 plays a direct role in cancer cell invasion by upregulating MMP protein expression. Mechanistically, ERK3 interacts with and phosphorylates steroid receptor coactivator-3 (SRC-3) at S857, which stimulates SRC-3-mediated MMP gene expression. SRC-3 is a nuclear receptor coactivator overexpressed in several types of cancers and considered a bona fide oncogene [58,59]. The phosphorylation of SRC-3 by ERK3 increases the interaction of SRC-3 with the transcription factor PEA3, leading to the upregulation of matrix metalloproteinase (MMP) gene expression (Figure 2) [36]. MMPs are secreted proteases that degrade the extracellular matrix and hence promote cell invasion [60]. Intriguingly, ERK3 mutations L290P and L290V confer increased migration and invasiveness on cancer cells [53]. As compared to wild type ERK3, ERK3^L290P^ and ERK3^L290V^ mutants have increased cytoplasmic localization and greater activity in promoting migration and invasion of several cancer cell lines.

In one of very few examples of protein inhibition, the diacylglycerol kinase DGKζ binds to the C34 domain of ERK3, preventing ERK3-facilitated migration and invasion in lung cancer cell lines [49]. Deletion of the C34 domain precluded both DGKζ interaction and the subsequent inhibition of ERK3-induced migration. These data join the growing body of work pointing to the C34 domain as an important region for protein interaction and regulation.

#### 3.2.3. ERK3 Confers Chemoresistance on Cancer Cells

Besides its role in cancer cell growth and invasiveness, several lines of evidence implicate ERK3 in the chemoresistance of cancer cells. In one study, ERK3 mRNA and protein levels were upregulated in doxorubicin-resistant MCF-7 breast cancer cells as compared to parental MCF-7 cells, suggesting the involvement of ERK3 in cancer chemoresistance [61]. Another study showed that ERK3 increased the chemoresistance of lung cancer cells to topoisomerase II inhibitors, such as etoposide, teniposide and doxorubicin, which induce DNA damage and apoptosis and are widely used as anti-cancer therapeutics [62,63]. Bian and colleagues showed that ERK3 interacted with and phosphorylated tyrosyl DNA phosphodiesterase 2 (TDP2), the component of the DNA repair pathway that repairs double-strand DNA breaks induced by topoisomerase II inhibitors [64]. This led to an increase in the phosphodiesterase activity of TDP2 and a concomitant decrease in the sensitivity of lung cancer cells to topoisomerase II inhibitors [62]. Further, a 2023 study demonstrated hepatocyte nuclear factor 4 gamma (HNF4G) binds to the ERK3 promoter and increases ERK3 protein level, increases AKT phosphorylation at S473 and reduces cisplatin sensitivity in lung adenocarcinoma cells [65]. An AKT inhibitor negated this protection, suggesting a role for the ERK3-AKT pathway in chemoresistance. Additional work is needed to fully understand the roles of ERK3 in chemoresistance in the context of different mutational settings and chemotherapies.

### 3.3. Tumor-Suppressing Roles of ERK3

Some studies have suggested that ERK3 has an inhibitory role in the growth, migration and invasion of cancer cells. In a tongue squamous carcinoma cell line, the exogenous expression of ERK3 inhibited migration and invasion, possibly by decreasing the level of Rac1 [66], which is important for lamellipodia extension and migration. In addition, ERK3 overexpression in these cells decreased cell proliferation by reducing the levels of several cell cycle regulatory proteins, transcription factors and growth factor receptors, including cdk1, cdk2, cdk4, cdk6 and JunB. Similarly, ERK3 inhibited migration, proliferation and colony formation in melanoma cells and a high ERK3 level was correlated with better survival of melanoma patients [67]. In the non-melanoma skin cancer cell line A431, ERK3 was identified to be a transcriptional target of ∆Np63α and mediated the inhibitory role for ∆Np63α in A431 cell migration [40]. However, ERK3 exhibited little effect on A431 cell proliferation. Moreover, overexpression of ERK3 in intrahepatic cholangiocarcinoma cells inhibited growth both in vitro and in vivo in xenograft mice [68]. This inhibitory effect on cell growth was concomitant with inactivation of mammalian target of rapamycin complex 1 (mTORC1), a major signaling component that affects cell survival in cholangiocarcinoma. Consistent with this, high ERK3 expression was associated with a better patient prognosis, as shown by analysis of ERK3 expression level in tumor samples resected from 73 patients with intrahepatic cholangiocarcinoma [68]. In line with these two studies, ERK3 decreased the proliferation of hepatocarcinoma cells but did not alter their migration [69]. Taken together, these studies report an inhibitory role for ERK3 on proliferation and/or migration of different cancer cell types.

In summary, these differential roles (either promoting or suppressing) identified for ERK3 suggest that it affects cancer cell growth and migration/invasion in a cancer type-dependent manner. The elucidation of the molecular mechanisms underlying the differential effects of ERK3 in cancer cells would entail greater knowledge about the substrates and the interacting partners of ERK3 in different cellular contexts.

## 4. Regulation of ERK3 in Cancers

**Figure 3:**
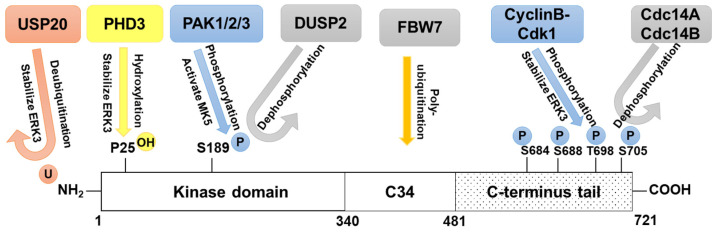
Post-translational modifications that regulate ERK3 stability and activity. Ubiquitination of the free N-terminus of ERK3 results in its degradation, while deubiquitination by USP20 stabilizes ERK3. Hydroxylation of P25 by PHD3 and phosphorylation of S684, S688, T698 and S705 by CyclinB-ckd1 stabilize ERK3 protein. The phosphatases Cdc14A and Cdc14B dephosphorylate ERK3. Phosphorylation of S189 by PAK1/2/3 results in activation of MK5, a well-characterized target of ERK3. DUSP2 dephosphorylates S189 in ERK3 and hence decreases MK5 activity. C34, conserved in ERK3 and ERK4; Cdc14A, cell division cycle 14A; DUSP2, dual-specificity phosphatase 2; FBW7, F-box/WD repeat-containing protein 7; MK5, MAPK-activated protein kinase-5; PAK, p21-activated protein kinases; PHD3, prolyl hydroxylase 3; USP20, ubiquitin-specific protease 20.

ERK3 activity, expression level and functions in cancers are regulated by a variety of mechanisms, including the regulation of ERK3 kinase activity, protein stability, subcellular localization and the regulation of mRNA transcript level (Figure 3).

### 4.1. Regulation of ERK3 Kinase Activity

The enzymatic activity of many protein kinases is regulated by phosphorylation(s) in the activation loop segment, which is located between subdomains VII and VIII of the kinase domain [70]. For example, dual phosphorylation of T185 and Y187 in the activation loop of the conventional MAPK ERK2 results in a dramatic increase in catalytic activity due to conformational changes that align the catalytic residues and the substrate binding site in optimal orientation [71]. The activation loop of ERK3, which lies between amino acids 174 and 216 and is highly conserved from zebrafish to human, is significantly similar in sequence and length to the activation loop of ERK1/2 [13,72]. However, the conserved TXY motif present in ERK1/2 is replaced in ERK3 by an SEG motif, which contains a single phosphorylation site, S189. The S189 residue in ERK3 corresponds to T185, the activating phosphorylation site in ERK2 [13,72]. In contrast to conventional MAPKs in which the activation motif is phosphorylated by an upstream MAPKK in response to extracellular stimulation, ERK3, when exogenously overexpressed, is constitutively phosphorylated at the single phospho-acceptor site S189 in the activation loop in resting cells, and this phosphorylation is not affected by exposure to mitogenic factors or cellular stresses [8,13,34]. In addition, phosphorylation of ERK3^S189^ can occur in *cis* as a result of autophosphorylation [3,8]. Interestingly, the C34 domain in the C-terminus is important for autophosphorylation [51]. ERK3 can also be phosphorylated on S189 in *trans* by the group I p21-activated protein kinases (PAKs) PAK1/2/3 [73,74]. The PAK family kinases are activated by the Rho GTPases Cdc42 and Rac. Phosphorylation of ERK3^S189^ by group I PAKs was simultaneously identified by two groups who used different approaches [73,74]. One group fractionated mammalian cell lysates and analyzed the proteins in fractions with high ERK3^S189^ phosphorylation, identifying PAK2 and PAK1 as potential kinase candidates. Further experiments demonstrated that PAK2 phosphorylates ERK3^S189^ in vitro, while both PAK2 and PAK3 increase ERK3^S189^ phosphorylation in cells [73]. The other group used active recombinant PAK2 to screen for potential substrates in high-density protein microarrays and identified ERK3. They confirmed the phosphorylation of ERK3 by PAK2 in vitro and identified S189 to be the phosphorylation site in ERK3 [74].

Following the identification of group I PAKs as kinases that phosphorylate ERK3^S189^, one study identified a member of the dual-specificity phosphatase (DUSP) family that dephosphorylates ERK3^S189^. By performing a yeast two-hybrid screen to identify potential interacting partners for ERK3 among the ten members of the mammalian DUSP family, Perander and colleagues revealed a specific interaction between ERK3 and DUSP2. This interaction, confirmed both in vitro and in mammalian cells at the endogenous level of both proteins, is dependent on the common docking (CD) domain in ERK3 and the kinase interaction motif (KIM) in DUSP2. Dephosphorylation of ERK3^S189^ by DUSP2 was demonstrated both in vitro and in cultured cells [75].

S189 phosphorylation has been shown to be important for ERK3 kinase activity and its cellular functions [5,9,13,50,73,75]. However, unlike the tight control of ERK1/2 activation by the dual phosphorylations of TXY motif, there is still insufficient evidence supporting a role for S189 phosphorylation in the SEG motif in controlling the activation of ERK3, likely due to limited knowledge of *bona fide* ERK3 substrates. Although MK5 is the best-characterized downstream target of ERK3 as of yet, the mechanism by which ERK3 stimulates MK5′s activating phosphorylation at Thr182 has yet to be clearly defined [10,11]. Debates remain as to whether ERK3 directly phosphorylates MK5 or only enhances MK5 autophosphorylation at Thr182 via physical interaction. Nevertheless, several groups have considered the phosphorylation of MK5 and its kinase activity as a readout for ERK3 activity, and hence they have investigated the importance of the phosphorylation of the activation loop of ERK3 by measuring MK5 activity [13,73,74]. These studies have demonstrated that the phosphorylation of ERK3^S189^ is absolutely required for binding to MK5 and for MK5 activation [13,74]. In line with these findings, the inhibition of group I PAKs in cultured cells reduced the phosphorylation of ERK3^S189^ and decreased the interaction between ERK3 and MK5 [74]. Also, group I PAK-mediated phosphorylation of ERK3 results in activation of MK5 [73], while DUSP2 decreases ERK3-mediated phosphorylation of MK5 in cultured cells [75]. Taken together, these studies strongly suggest that S189 phosphorylation of ERK3 is critical for the interaction between ERK3 and MK5 and ERK3-mediated MK5 activation. However, it would be interesting to determine whether the S189 phospho-site is only involved in substrate binding or also directly regulates the catalytic activity of ERK3.

To understand how S189 phosphorylation is important for the binding of ERK3 to MK5, even though the interaction between ERK3 and MK5 is mediated by FRIEDE docking motif in ERK3, Aberg and colleagues used molecular modeling. They showed that ERK3^S189^ phosphorylation increases the surface exposure of I334 residue within the FRIEDE motif, which thus facilitates the interaction with MK5 [12].

### 4.2. Molecular Regulation of ERK3 Expression

Various molecular mechanisms by which the expression of ERK3 is regulated have been recently described. Interestingly, several studies have revealed a regulatory cross-talk between the activation of conventional MAPKs and ERK3 expression. For example, the study by Hoeflich and colleagues showed that the transcription of ERK3 gene (*MAPK6*) was upregulated by the Ser/Thr protein kinase B-Raf [76], a MAPKKK that is an essential component of ERK1/2 pathway. Pharmacological inhibition of the MAPKKs MEK1/2, which are downstream of B-Raf, resulted in a decrease in ERK3 expression, suggesting that the regulation of ERK3 by B-Raf was mediated by MEK1/2 [76]. A later study confirmed BRAF upregulated ERK3 in a kinase-dependent manner [67]. In line with these studies, both overexpression and constitutive activation (G12C) of KRAS, an upstream activator of BRA/MEK1/2/ERK1/2 signaling cascade, stimulate ERK3 expression level in NSCLCs [44].

Direct regulators of ERK3 gene transcription have also been identified. c-Jun was shown to mediate ERK3 gene transcription upon the stimulation by the cytokines TNF-α and IL-1β in endothelial cells [21]. Later studies agreed TNF-α and IL-1β are inducers of ERK3 [22,33]. Besides induction by cytokines, prolactin hormone was shown to stimulate ERK3 mRNA expression as well, but it is unclear which transcription factor(s) is involved in this regulation [29]. In addition, ΔNp63α, a transcription factor which is upregulated in non-melanoma skin cancer, binds directly to two specific sequences in the ERK3 promoter region and is correlated with higher ERK3 expression [40]. Moreover, TATA-box-binding protein-associated factor 15 (TAF15) was reported to be recruited to ERK3 mRNA via the long non-coding RNA (lncRNA) LINC00649 in lung squamous cell carcinoma and via the circular RNA (circRNA) circDNAJC11 in breast cancer cells and upregulate ERK3 mRNA level, likely by increasing ERK3 mRNA stability [77,78]. In an example of negative regulation, ZNF671 reduced MAPK6 promoter activity and ERK3 level in the laryngeal carcinoma cell lines AMC-HN-8 and TU177 [39].

Besides the transcriptional regulation, several recent studies have identified post-transcriptional regulation of ERK3 level by microRNAs (miRNAs). MiRNAs are endogenous non-coding RNAs that bind to target mRNAs and lead to translational repression and/or mRNA degradation; hence, they act as important post-transcriptional regulators of gene expression [79]. Considering the short half-life of ERK3, microRNA regulation is a potentially central platform for the regulation of this protein. Multiple studies have linked ERK3 expression to specific miRNAs. In a study using head and neck cancer cells, an axis comprising the polycomb group protein BMI1 and the miRNA let-7i was shown to regulate the expression of ERK3 [52]. The miRNA let-7i, which is downregulated in several cancers and decreases cancer cell migration and invasion [80,81,82], decreased ERK3 protein level by directly targeting the 3′UTR of ERK3 mRNA [52]. BMI1 is a major component of the transcription suppressor complex polycomb repressive complex 1 (PRC1) and is an oncogenic protein [83]. Through inhibiting the transcription of let-7i, BMI1 positively regulated ERK3 level [52]. LncRNAs may also play a role in ERK3 expression, as evidenced by the TAF15 data noted above. In addition, the lncRNA nuclear enriched abundant transcript 1 (NEAT1) facilitates ERK3 expression by absorbing miR-98-5p, which targets ERK3 expression [84,85].

### 4.3. Regulation of ERK3 Stability

ERK3 is unique among the MAPK family for being a highly unstable protein with a half-life of about 30–60 min in proliferating cells [34,35], signifying that it can be acutely regulated by protein turnover. Several post-translational modifications (PTMs), including ubiquitination, hydroxylation and phosphorylation, play important roles in the regulation of ERK3 protein stability. Ubiquitination of ERK3 is required for its proteolysis by the 26S proteasome. Notably, ubiquitination and degradation of ERK3 are not dependent on activation loop phosphorylation, kinase activity or the presence of the C-terminus tail [34]. Unlike the majority of ubiquitinated proteins in which ubiquitin is conjugated to an internal lysine residue, ubiquitin is conjugated to the free N-terminus of ERK3. Hence, addition of an N-terminal tag larger than 5 kDa on ERK3 stabilizes it by hindering the ubiquitination process [86]. FBW7, an F-box protein that is part of the S-phase kinase-associated protein 1 (SKP1)-cullin 1-F-box protein (SCF) E3-ubiquitin ligase for protein degradation, binds ERK3 to promote its degradation through ubiquitin [48]. The T417 and T421 residues in the C34 domain act as a degron motif to facilitate ERK3 interaction with FBW7, but the actual site(s) for ubiquitination by SCF-FBW7 remains to be identified.

Opposite to E3-ligase-mediated ubiquitination and degradation, deubiquitinases, such as the ubiquitin-specific proteases (USPs), remove ubiquitination and stabilize proteins [87]. One study showed that USP20 deubiquitinates ERK3 both in vitro and in cultured cells, leading to its stabilization [35]. The interaction between USP20 and ERK3 was demonstrated at endogenous levels of both the proteins in cultured cells. Consistent with the regulation of ERK3 protein by USP20, a strong correlation was shown between their expression levels in various cellular contexts. In addition, a novel role for USP20 in promoting actin cytoskeleton organization and cell migration was revealed and linked to its regulation of ERK3 expression. Two other DUBs, USP13 and USP16, also regulate the levels of ERK3 in cultured cells, albeit their effect is modest [35].

Besides being stabilized by deubiquitination, ERK3 stability is increased during mitosis by phosphorylation of its C-terminus tail [45]. Analysis of ERK3 expression at different stages of the cell cycle revealed that ERK3 protein accumulates in mitotic cells, concomitant with its hyperphosphorylation, which is reversed during M → G_1_ transition. By in vitro incubation of recombinant ERK3 protein with mitotic cell extracts, phosphorylation at four residues located in the C-terminal tail of ERK3 was identified. The phosphorylation of ERK3 on one or more of these four sites (Ser684, Ser688, Thr698 and Ser705) results in its stabilization. Phosphorylation of ERK3^T698^ and ERK3^S705^ was confirmed in cultured cells during mitosis [45]. ERK3^T698^ is phosphorylated by cyclin B-cyclin-dependent kinase 1 (Cdk1), which is required for normal entry and progression into mitosis [45,88], while the phosphatases cell-division cycle 14A (Cdc14A) and Cdc14B, which are implicated in mitotic exit, interact with and dephosphorylate ERK3 in vitro and reverse ERK3^T698^ phosphorylation in a cellular context [45,88]. While the molecular mechanisms underlying the phosphorylation-dependent stability increase of ERK3 during mitosis await elucidation, this finding is in agreement with its important roles in mitosis and cytokinesis as discussed above [43,47,48].

Another PTM that regulates ERK3 stability is hydroxylation. The interaction between ERK3 and prolyl hydroxylase 3 (PHD3) was identified by a quantitative interaction proteomics screening for PHD3 substrates and confirmed in cells at endogenous levels of the two proteins [89]. By mass spectrometry, it was found that PHD3 hydroxylates Pro25 in ERK3, protecting ERK3 from proteasomal degradation. This was demonstrated by the findings that hydroxylase inhibitors promoted ERK3 degradation and that a mutant ERK3^P25A^ was expressed at lower level than the wild type, and its level was not further suppressed by hydroxylase inhibitors. Egl-9 family hypoxia-inducible factor 3 (EGLN3)-facilitated hydroxylation also promotes ERK3 protein levels by preventing interaction with proteins in the chaperone-mediated autophagy (CMA) pathway [90].

ERK3 protein stability has also been shown to be regulated by other proteins, although the exact underlying mechanisms are yet to be determined. Several studies observed that the interaction between ERK3 and MK5 results in cytoplasmic localization and stabilization of both proteins [10,11,12]. In addition, Rab31, a small-molecule GTP-binding protein that regulates protein transport in the trans-Golgi network, was reported to have physical interaction with ERK3 and increases its protein stability [54]. Rab31 and ERK3 cooperatively regulate cytoskeletal rearrangement and promote cell motility. Given that the ERK3/MK5 axis exhibits a similar role in regulating cytoskeletal structure and cell motility, it would be interesting to determine whether Rab31 has similar effect on MK5.

### 4.4. Regulation of ERK3 Localization

Modulating spatiotemporal localization of proteins is one mechanism by which cells can regulate access to interacting partners and substrates. In line with its various roles in cellular processes, ERK3 is localized to different cellular compartments, including the plasma membrane [49,55,57], cytoplasm and nucleus [8,53], as well as the golgi/endoplasmic reticulum [91]. When tagged with a nuclear localization sequence, ERK3 had enhanced nuclear localization, resulting in increased cell proliferation compared with the wild type ERK3 [92]. ERK3 interacts with MK5, facilitating the cytoplasmic localization and stabilization of both proteins [13]. In 3T3 cells, inhibiting group I PAKs led to increased ERK3 in the nucleus, and in 293T cells, PAK inhibition led to a reduction in ERK3 interaction with MK5 [74]. CRM1, a nuclear export protein, interacts with ERK3 to facilitate its nuclear export [92]. ERK3 deletion mutant experiments suggest the nuclear export sequence is between amino acids 399–452. Chimeric experiments with ERK2 and ERK3 suggest the second half of the ERK3 kinase domain is important for its nuclear localization [93].

ERK3 cytoplasmic localization and translocation to plasma membrane protrusions may be important for its role in regulating cytoskeletal structure and cell motility. ERK3 colocalizes with Cdc42 and Rac1 in membrane protrusions at the leading edge of cells [57,94] and increases their activity towards PAK1 kinase, thereby upregulating ARP2/3-mediated F-actin polymerization and cell motility. Interestingly, L290P/V mutations of ERK3 that are identified in some cancers, albeit at a low frequency, cause an increase in cytosolic localization of ERK3 and enhanced ability to promote migration and invasion in lung cancer cell lines [53]. Thus, it will be interesting to determine whether ERK3L290P/V mutants have increased interaction with Cdc42 and Rac1 and/or enhanced activity in upregulating Cdc42 (Rac1)/PAK1/Arp2/3 signaling, which may account for its increased ability to promote cell migration and invasion [49,55,57]. ERK3 can also localize to the Golgi/endoplasmic reticulum, suggesting a potential role in protein trafficking [91]. In relation to this, ERK3 was found to interact with Rab31, a GTP-binding protein known to regulate protein transport from Golgi to plasma membrane [54].

## 5. Conclusions and Perspective

There have been major advances in our understanding of the functions and mechanistic regulation of ERK3 in recent years. In particular, our insights into the roles for ERK3 in cancer development progression are making significant headway. Of note, accumulating studies, including some showing contradicting findings, have suggested that ERK3 plays differential or context-specific roles in different types (or subtypes) of cancer. However, there is a need for further investigation to uncover molecular mechanisms underlying the differential roles of ERK3, which might include different genetic mutations, dysregulation of other signaling pathways and different tumor microenvironments. The C34 domain appears to be an important region for protein docking. A growing body of studies have identified microRNA regulators of ERK3 level, helping to contextualize ERK3 in known and novel pathways. Physiologically, our understanding of ERK3′s role in inflammation and obesity has been expanded. Furthermore, recent studies have shed light on the role of PTMs in regulating the stability of ERK3. However, many gaps in knowledge remain, including the identity of upstream activators of ERK3 and downstream substrates. The recent development of ERK3 inhibitors [95] may offer new experimental methods for studying ERK3. While future studies will eventually solidify ERK3′s roles in cancer cell motility and growth, clarify the underlying mechanisms and set these firmly in the backdrop of ERK3′s physiological roles, considerable work is needed to reach this point, with many questions currently unanswered.
